# Dynamic and Static Effects of the Systemic Inflammatory Response Index on All-Cause Mortality in Individuals With Atherosclerotic Cardiovascular Disease: Evidence From National Health and Nutrition Examination Survey

**DOI:** 10.1155/mi/5343213

**Published:** 2025-04-16

**Authors:** Chenglin Duan, Yihang Du, Jiafan Chen, Shuqing Shi, Xiaohan Zhang, Yuanhui Hu

**Affiliations:** ^1^China Academy of Chinese Medical Sciences Guang'anmen Hospital, Beijing 100053, China; ^2^Graduate School of Beijing University of Chinese Medicine, Beijing 100029, China

**Keywords:** all-cause mortality, atherosclerotic cardiovascular disease, mediation, serum uric acid, systemic inflammatory response index

## Abstract

**Objective:** This research focuses on analyzing the link between the systemic inflammatory response index (SIRI) and all-cause mortality in individuals with atherosclerotic cardiovascular disease (ASCVD) .

**Methods:** This research analyzed data from 4693 patients using nine cycles of the National Health and Nutrition Examination Survey (NHANES). The connection between SIRI and mortality was determined by employing survey-weighted Cox models, with hazard ratios (HRs) and 95% confidence intervals (CIs) being computed. Kaplan–Meier method illustrated survival differences across SIRI levels. Sensitivity analyses involved restricted cubic splines (RCS), stratified analysis, and *E*-value calculations. Landmark analysis assessed survival differences at multiple follow-up intervals, while time-dependent receiver operating characteristic curves evaluated SIRI's prognostic value. Mediation analysis identified potential intermediaries impacting the SIRI–mortality relationship.

**Results:** Over 406,564 person–months, 1933 deaths occurred. Adjusted Cox models discovered that higher SIRI was connected with elevated overall mortality [HR 1.192, (95% CI 1.131–1.256), *p* < 0.001]. Higher SIRI consistently showed lower survival probabilities. RCS and stratified analysis confirmed the robustness of these findings. Survival probability at different follow-up periods was considerably lower in those with higher SIRI. Additionally, SIRI demonstrated a prognostic value of 0.66 for all-cause mortality at 1 year and 3 years, and 0.65 at 5 years. Notably, serum uric acid (6.2%) partially mediated the connection between SIRI and mortality from all causes.

**Conclusion:** In ASCVD patients, SIRI was robustly correlated with all-cause mortality, partially mediated by serum uric acid.

## 1. Introduction

Cardiovascular diseases constitute the leading cause of death worldwide, responsible for ~32% of all deaths [1]. The predominant contributors to this statistic are ischemic heart disease and stroke [1, 2]. Among the various forms of CVD, atherosclerotic cardiovascular disease (ASCVD) is the most prevalent. It is characterized by the build-up of atherosclerotic plaques in the arterial walls [2, 3]. These plaques cause a gradual narrowing of arteries, hindering movement to various organs and tissues. The development of ASCVD is driven by a complex interaction of genetic, environmental, and lifestyle influences [4–6].

Inflammation is increasingly acknowledged as an essential element in the onset and advancement of ASCVD [7]. The role of chronic low-grade systemic inflammation in ASCVD is particularly significant, serving as a primary driver of atherosclerosis by promoting endothelial dysfunction, lipid accumulation, and plaque formation [7, 8]. Epidemiological data strongly indicate a connection between high levels of inflammatory biomarkers and the elevated ASCVD risk. Notably, biomarkers like tumor necrosis factor-α (TNF-α), C-reactive protein, and interleukin-6 (IL-6) are robustly linked to higher occurrences of stroke, coronary heart disease, and death from cardiovascular disease [9–11].

Inflammation in cardiovascular diseases has spurred significant interest in developing biomarkers that can accurately gauge the underlying inflammatory state and forecast adverse cardiovascular events [12]. Among these, the systemic inflammatory response index (SIRI) stands out. This index, integrating neutrophil, lymphocyte, and monocyte levels, is a promising indicator for evaluating systemic inflammation [13]. Demonstrating reliability in reflecting systemic inflammation, SIRI is connected to poor prognoses in various cardiovascular disorders, including ischemic stroke and coronary artery disease [14, 15].

Despite valuable insights from previous research, the exact effect of SIRI on overall mortality in individuals with ASCVD is still ambiguous. Striving to address this gap, this research examines the future correlation between SIRI and overall mortality in individuals diagnosed with ASCVD, utilizing representative population data. Additionally, this study examines potential mediators affecting the connection between SIRI and overall mortality risk among this patient cohort.

## 2. Methods and Materials

### 2.1. Data Source

Participants for this research were drawn from the National Health and Nutrition Examination Survey (NHANES) database, which is administered by United States government agencies and provides a national representation. NHANES focuses on examining the state of health and nutrition of the American residents. A complex, stratified, and multistage sampling strategy is adopted for achieving a representative sample of noninstitutionalized American citizens. NHANES collects data biennially on participants' demographic characteristics, physical measurements, blood test parameters, health conditions, and nutritional status, with collection performed by trained personnel. The NHANES program has undergone rigorous review and received approval from the NCHS Research Ethics Review Board. The NHANES program ensures that all individuals enroll voluntarily and provide written informed consent. To ensure confidentiality, NHANES professionals employ various methods to anonymize identifiable patient characteristics, thereby maintaining participants' privacy throughout this research.

### 2.2. Study Population

**Figure 1:**
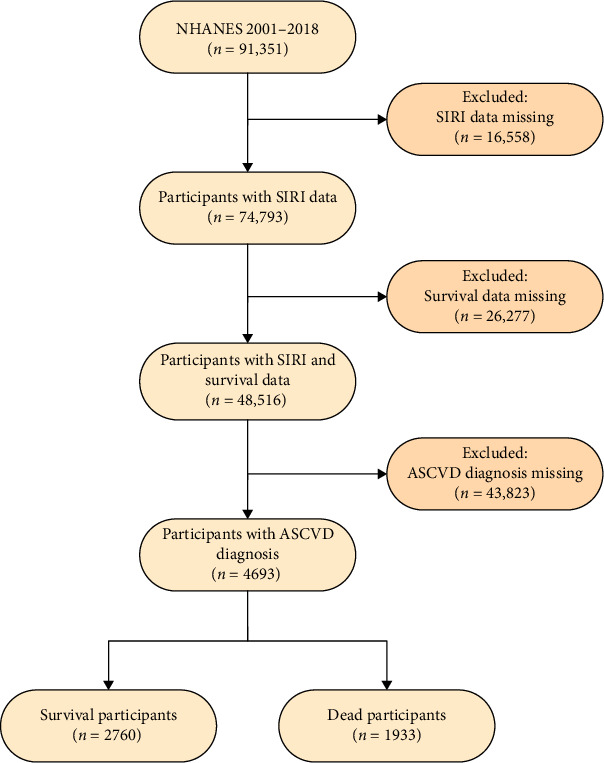
Flowchart of patient enrollment.

A sum of 91,351 participants across nine NHANES survey cycles, from 2001–2002 to 2017–2018, were initially recruited for this study. According to the 2013 ACC/AHA guidelines, ASCVD is characterized by one or more concerning the conditions below: stroke, angina pectoris, heart attack, and coronary heart disease [16]. In this investigation, ASCVD data were collected by NHANES staff through interviewer–administered questionnaires. Individuals were classified as having ASCVD based on self-reports of the specified conditions during the health status interview. To secure the precision and steadiness of the study discoveries, stringent exclusion criteria were applied. Participants were excluded for (1) missing data on neutrophil, lymphocyte, or monocyte counts (*n* = 16,558); (2) incomplete survival status records (*n* = 26,277); or (3) not meeting the ASCVD diagnostic criteria (*n* = 43,823). Following these exclusions, the final study population comprised 4693 participants (Figure 1).

### 2.3. Calculation of SIRI

Blood samples were entirely collected and processed at the NHANES Mobile Examination Centre, adhering to standardized protocols to ensure consistency and accuracy across all measurements. Detailed methodologies for specimen acquisition and preparation are thoroughly explained in the NHANES Laboratory/Medical Technologists Procedures Manual. The analysis of participants' peripheral blood samples was conducted using the Beckman Coulter MAXM instrument. This instrument is extensively validated and commonly employed for cell counting in both clinical and research environments. The cell counts were reported in units of 1000 cells/µL, a standard unit that enhances comparability across various studies and populations. The SIRI calculation involved the product of neutrophil and monocyte counts divided by the lymphocyte count [13].

### 2.4. Ascertainment of Mortality Outcomes

The survival status of participants was rigorously assessed using the linked mortality records file from the NHANES database, connected to the National Death Index. This linkage issued a reliable method for verifying the survival status of participants and identifying causes of death. To guarantee precise and comprehensive mortality data, participants were monitored until either December 31, 2019, or their date of death, whichever took place first. Any death, regardless of cause, was specified as all-cause mortality. Detailed information regarding participants' survival status is available through the Centres for Disease Control and Prevention at https://www.cdc.gov/nchs/data-linkage/mortality-public.html.

### 2.5. Covariates

The current study accounted for a variety of covariates to ensure a robust analysis, including demographic characteristics, lifestyle factors, physical measurements, and past medical history. The study considered demographic factors such as gender, age, poverty-income ratio (PIR), education, and race/ethnicity. The classification for race/ethnicity included “White,” “Black,” “Mexican,” and “other.” Education was categorized as “<high school,” “high school,” or “>high school.” Lifestyle factors encompassed smoking status and alcohol consumption. For alcohol, individuals were divided into non-drinkers (consumed < 12 drinks in lifetime), former drinkers (consumed ≥ 12 drinks but abstained in the past year), and current drinkers (consumed ≥ 2 drinks monthly for men and ≥ 1 drink for women in the past year) [17]. Similarly, smoking status was classified as nonsmokers (consumed < 100 cigarettes in lifetime), former smokers (consumed ≥ 100 cigarettes but had quit), and current smokers (consumed ≥ 100 cigarettes and continues to smoke) based on lifetime smoking status and current smoking status [18]. The body mass index (BMI) was one of the physical measurements. Past medical history variables focused on type 2 diabetes, hypertension, and hyperlipidemia.

### 2.6. Statistical Analysis

The intricate NHANES sampling methodology was meticulously accounted for in this study, incorporating participant weights from 2001 to 2018 into the statistical analysis [19, 20]. To address the potential loss of data from missing covariate values, multiple imputation techniques were employed to fill these gaps. To compare categorical variables, the chi-square test was used, while the Wilcoxon test was applied to continuous variables.

For covariate evaluation in the study, we used both the Boruta algorithm and univariate Cox regression. Cox proportional hazards models were implemented, examining SIRI as both a continuous and categorical variable to ascertain 95% confidence intervals (CIs) and hazard ratios (HRs). Adjustments for covariates were consistently applied across all models: Model 1 remained unadjusted; Model 2 was adjusted for gender, age, education, race/ethnicity, and PIR; Model 3 included adjustments for lifestyle factors like BMI, alcohol consumption, and smoking status; and Model 4 also incorporated adjustments for health conditions including hypertension, type 2 diabetes, and hyperlipidemia. Using the weighted restricted cubic spline (RCS) method, the study investigated the potential nonlinear connection of SIRI with survival outcomes in ASCVD patients. Stratified analyses based on demographic and medical history variables, such as age, gender, race, BMI, and histories of type 2 diabetes, hypertension, and hyperlipidemia, were conducted. The influence of potential unmeasured confounding factors was estimated by calculating the *E*-value [21].

Using Kaplan–Meier curves, the survival probabilities for ASCVD patients at different SIRI levels were depicted, and the log-rank test evaluated survival differences. Additionally, Kaplan–Meier curves were plotted using the "jskm" software package at 1, 3, and 5 years to evaluate survival probability differences at these intervals. Time-dependent receiver operating characteristic (ROC) curves were formulated to estimate the prognostic utility of SIRI for survival status at various time points.

To delve deeper into the mediating factors influencing the connection between SIRI and survival outcomes, factors such as oxidative stress markers (*γ*-glutamyl transferase, total bilirubin, and serum uric acid) were considered [22]. Using R software (version 4.3.1), all statistical analyses were carried out, applying two-sided tests with a *p* − value cut-off of 0.05.

## 3. Results

### 3.1. Baseline Characteristics of Study Population

In total 4693 participants from nine NHANES cycles were incorporated into this study (Figure 1). Over the follow-up period, which spanned 406,564 person–months, 1933 participants (41.19%) were recorded as deceased. The baseline parameters of ASCVD individuals are classified by survival status (Table 1). The deceased cohort was generally older and included more individuals of White race than the survival group. They also exhibited lower levels of educational attainment and income. Notably, the deceased group had higher histories of smoking and alcohol consumption, lower BMI, and lymphocyte counts, alongside higher neutrophil counts. Moreover, the occurrence of type 2 diabetes and hypertension was markedly higher among the deceased cohort, highlighting the impact of chronic disease burden on mortality rates among ASCVD patients.

### 3.2. Relationship Between SIRI and Survival Outcome

Using data from the Boruta algorithm and univariate Cox regression analysis, we incorporated various covariates into our study, involving gender, age, PIR, education, race/ethnicity, BMI, alcohol consumption, smoking status, and past medical history (Figure S1 and Table S1). The unadjusted model showed that elevated SIRI was connected to a higher risk of overall mortality in ASCVD individuals [HR 1.272, (95% CI 1.192–1.356), *p* < 0.001]. The multivariable-adjusted models offered further insights into the connection between SIRI and mortality. According to Model 2, each unit elevates in SIRI was related to a 19.9% increased risk of death (HR 1.199, [95% CI 1.135–1.267], *p* < 0.001). Model 3 revealed a 19.6% elevate in mortality risk for every unit increment in SIRI (HR 1.196, [95% CI 1.137–1.259], *p* < 0.001). Similarly, Model 4 demonstrated that with each incremental unit elevates in SIRI, the likelihood of death rose by 19.2% (HR 1.192, [95% CI 1.131–1.256], *p* < 0.001) (Table 2). The findings emphasize a robust linear connection between increased SIRI and greater risk of death among ASCVD individuals. The *E*-value of 1.519, exceeding the corresponding HR, underscores that the influence of unmeasured confounders is minimal, thus affirming the credibility of our findings.

**Figure 2:**
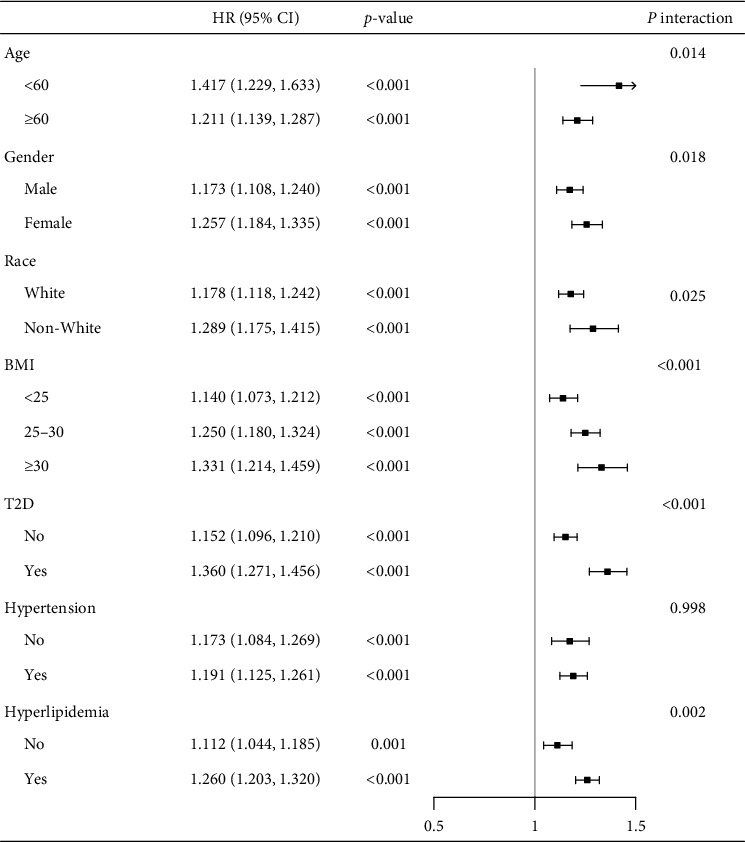
Stratified analyses of the relationship between SIRI and all-cause mortality. BMI, body mass index; CI, confidence interval; HR, hazard ratio; SIRI, systemic inflammatory response index; T2D, type 2 diabetes.

To gain deeper insights, stratified analyses were carried out, examining the connection between SIRI levels and all-cause mortality risk among various demographic and clinical subgroups. We examined subgroups based on age (<60 or ≥60 years), gender, race, BMI ranges (<25, 25–30, or ≥30 kg/m^2^), and histories of type 2 diabetes, hyperlipidemia, and hypertension. Within ASCVD individuals in each subgroup, a notable positive correlation was detected between higher SIRI levels and overall mortality risk (all *p* < 0.05) (Figure 2). Stratified analysis results support the general conclusions, demonstrating the robustness of the connection in different patient subgroups. Notably, except for hypertension history, significant interactions between SIRI and the risk of death from all causes were detected in all stratified factors (*p* for interaction <0.05 for all except hypertension). These findings further underscore the consistency and reliability of SIRI as a prognostic marker across diverse clinical profiles.

According to their quartile levels of SIRI, participants were categorized into four groups. The survival probabilities for groups with higher SIRI levels were significantly lower, as shown by the survival curves, when contrasted with those in lower quartiles (*p* for log-rank test < 0.001) (Figure S2). Participants in higher SIRI quartiles experienced a progressively higher mortality risk relative to individuals in the lowest quartile, as shown in both unadjusted and multivariable-adjusted Cox analyses, indicating a clear linear positive pattern (*p* for trend < 0.001) (Table 2). The evidence additionally supports the significant connection between elevated SIRI and increased mortality from all-causes among ASCVD individuals, emphasizing SIRI's usefulness as a prognostic tool.

**Figure 3:**
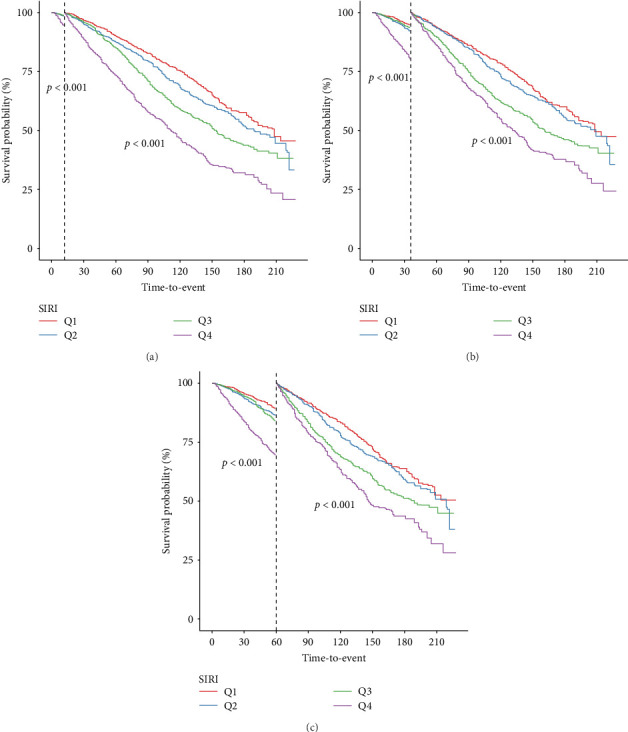
Kaplan–Meier survival curves for all-cause mortality at (A) 1 year, (B) 3 years, and (C) 5 years with landmark method. SIRI, systemic inflammatory response index.

Landmark analysis was engaged to investigate the variations in survival probabilities at specific intervals—after 1, 3, and 5 years—among groups categorized by SIRI levels (Figure 3). Kaplan–Meier analysis confirmed that at each time marker—1, 3, and 5 years—the survival probability for the group with higher SIRI levels remained consistently inferior to those for the group with lower levels, across all specified times. Importantly, these differences in survival rates between the groups were statistically significant at all three-time points, indicating a robust relationship between elevated SIRI levels and decreased survival across multiple intervals.

The correlation between SIRI levels and mortality from all causes among ASCVD individuals is illustrated in Figure S3 through a dose–response connection. The RCS analysis, after adjusting for covariates, mapped a linear connection between escalating SIRI and the risk of mortality from all causes in ASCVD participants. Additionally, the analysis discovered a meaningful nonlinear aspect within this connection (nonlinear *p*=0.025). This suggests a complex interaction between systemic inflammation, as quantified by SIRI, and mortality risk, underscoring the nuanced impact of inflammation on health outcomes.

### 3.3. Prognostic Value Assessment

The consequences of the time-dependent ROC analysis are displayed in Figure S4, depicting the areas under curve (AUC) for SIRI as predictors of all-cause mortality. At 1 year, the AUC stood at 0.66, remained 0.66 at 3 years, and slightly decreased to 0.65 at 5 years. The data suggest that SIRI provides reliable prognostic utility for determining risk of death from all causes among ASCVD individuals throughout these time spans.

### 3.4. Mediation Analyses

**Figure 4:**
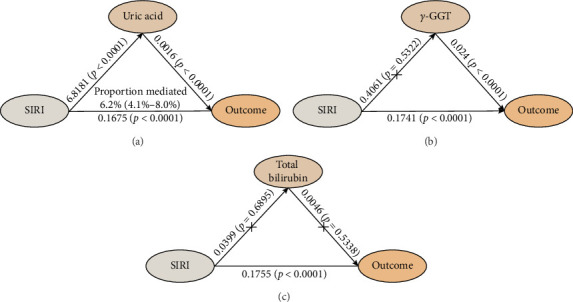
The mediating effects of (A) serum uric acid, (B) *γ*-GGT, and (C) total bilirubin on the relationship between SIRI and survival outcome. *γ*-GGT, gamma-glutamyl transferase; SIRI, systemic inflammatory response index.

The study analyzed the effects of oxidative stress markers in mediating the connection between SIRI and mortality risk from all causes. SIRI had a positive correlation with serum uric acid (*β* ± SE = 6.8181 ± 1.6731, *p* < 0.0001) (Figure 4). Additionally, the correlations between SIRI and γ-glutamyl transferase (*β* ± SE = 0.4061 ± 0.6483, *p*=0.5322) and total bilirubin (*β* ± SE = 0.0399 ± 0.0996, *p*=0.6895) were not statistically significant (Figure 4). Mediation analysis revealed that serum uric acid mediated 6.2% (95% CI 4.1%–8.0%) of the connection between SIRI and risk of death from all causes (Figure 4). These results highlight the significant roles that these biomarkers play in the pathway between systemic inflammation, as measured by SIRI, and mortality outcomes in ASCVD patients.

## 4. Discussion

Using data from 4693 ASCVD patients in the NHANES database, this study established an independent connection between SIRI and death from all causes in this patient group. The results consistently demonstrated a linear positive interrelation between SIRI levels and risk of death from all causes, a finding that persisted across various sensitivity analyses. By categorizing patients into quartiles based on their SIRI levels, both Cox regression and survival curve analyses revealed that higher SIRI significantly correlates with greater mortality risk. Additional analyses at landmark intervals—1, 3, and 5 years—consistently indicated a connection between higher SIRI levels and greater risk of death from all causes in ASCVD individuals. Time-dependent ROC analysis affirmed the prognostic efficacy of SIRI for survival outcomes at these intervals. Additionally, mediation analysis indicated that serum uric acid (6.2%) partly mediated the impact between SIRI and mortality risk. These insights highlight the potential of using SIRI as a prognostic tool to early identify systemic inflammatory status in ASCVD patients, aiming to decrease the risk of premature death in this group. The insights garnered from this study underscore the prognostic value of SIRI and point toward its utility in clinical assessments and targeted interventions aimed at managing inflammation-driven mortality risks in ASCVD.

Epstein [23] posited that atherosclerosis is essentially a chronic, low-grade inflammatory condition affecting the arteries, significantly underscoring the pivotal role inflammation plays in initiating and advancing atherosclerosis. This perspective explains how various risk factors lead to arterial endothelial cell dysfunction, resulting in heightened permeability, increased levels of cell adhesion proteins, and elevated release of chemokines and inflammatory substances. These changes facilitate the adhesion and migration of inflammatory cells, triggering localized inflammatory responses [24]. Compounding the issue, dysfunction in endothelial cells stimulates the excretion of inflammatory cytokines, such as TNF-*α* and IL-6. These substances further damage the endothelium and sustain a harmful cycle [10, 25]. The increased permeability of endothelial cells allows low-density lipoprotein to infiltrate the vascular intima, where it oxidizes into oxidized low-density lipoprotein [26]. Oxidized low-density lipoprotein is particularly damaging because it triggers activation in endothelial cells and macrophages, promotes increased levels of adhesion proteins and chemokines, and enhances the recruitment of monocytes, thereby amplifying the inflammatory response [27]. As atherosclerotic plaque formation advances, the influx of inflammatory cells, increased levels of inflammatory mediators, and the complex interactions between cytokines and chemokines together enhance the growth and maturation of these plaques. ASCVD is characterized by persistent, low-grade chronic inflammation, often accompanied by the release of inflammatory cytokines, such as IL-6 and TNF-*α*, in response to tissue damage [28]. Elevated levels of the SIRI are typically indicative of increased systemic inflammation, which may further enhance the expression of various inflammatory mediators. TNF-*α* contributes to endothelial cell damage, increasing permeability, and promoting lipid deposition in the vessel walls, leading to the formation of atherosclerotic plaques [26]. Additionally, TNF-*α* suppresses lipoprotein lipase activity, hindering lipid breakdown, and further advancing plaque progression [26]. IL-6 plays a key role in promoting the release of inflammatory mediators, amplifying the inflammatory response, and contributing to endothelial dysfunction, which accelerates plaque formation and progression [29]. Furthermore, IL-6 is associated with plaque instability, microvascular dysfunction, and adverse outcomes in acute ischemic events [30, 31]. This comprehensive understanding underscores the inflammatory underpinnings of atherosclerosis, illustrating the pivotal role of inflammation in this vascular disease.

Known as white blood cells, immune cells are vital throughout the development of atherosclerosis, from its onset to its advancement. These cells mainly consist of three subtypes: granulocytes, monocytes, and lymphocytes. Among the early responders, monocytes quickly infiltrate areas affected by atherosclerosis. When stimulated by various cytokines, endothelial cells trigger local inflammatory responses in the arterial vessel wall, resulting in increased production of chemokines and adhesion molecules. This mechanism promotes the adhesion of circulating monocytes to endothelial cells and their migration into the subendothelial space, where they differentiate into macrophages [32]. Macrophages drive the progression of atherosclerosis through multiple pathways: (1) absorb oxidized low-density lipoprotein, transforming into foam cells that help expand the lipid core within plaques; (2) generate reactive oxygen species, intensifying local oxidative stress; and (3) excrete inflammatory cytokines like TNF, IL-1, and IL-6, exacerbating the inflammatory response within the arterial vessel wall [32]. T lymphocytes, particularly T helper cell 1, are critical in the development of atherosclerosis. Predominant in early plaque stages, these cells secrete interferon-*γ* [33, 34], which not only promotes macrophage differentiation towards the M1 phenotype, inducing the production of major histocompatibility complex II and inflammatory cytokines but also stimulates vascular endothelial cells to produce chemokines, enhancing T cell infiltration [33–36]. Neutrophils contribute significantly in the initial stages of atherosclerosis. They upregulate endothelial adhesion molecules and modulate permeability, facilitating the recruitment and adhesion of additional immune cells [37]. Upon activation, neutrophils upregulate various integrins and release reactive oxygen species, myeloperoxidase, neutrophil elastase, and matrix metalloproteinases, all of which expedite atherosclerosis progression. Additionally, the formation of neutrophil extracellular traps also contributes to the disease [38]. Overall, the interplay of neutrophils, monocytes, and lymphocytes illustrates the comprehensive immune involvement in the formation and progression of atherosclerosis, highlighting their crucial roles in the localized inflammatory responses that characterize this disease.

Although direct evidence linking SIRI directly to all-cause mortality in ASCVD patients remains limited, its relationship with various subtypes of ASCVD has been documented. A notable cross-sectional study involving 699 individuals from a Polish population demonstrated a clear link between elevated SIRI and the degree of coronary artery disease severity [14]. Similarly, Zhang et al. [15], using data from 2450 acute stroke patients in the MIMIC-III database, found an important correlation between SIRI and mortality from all causes in this population. Importantly, this study demonstrated that SIRI had superior prognostic accuracy for mortality compared to other blood cell-derived inflammatory markers, including the lymphocyte-to-monocyte, platelet-to-lymphocyte, and neutrophil-to-lymphocyte ratios [15]. Additionally, another study involving 310 patients who had experienced acute myocardial infarction—81 of whom faced major adverse cardiovascular events—showed that SIRI levels were indicative of the likelihood of such events occurring, even after adjusting for various confounding factors [39]. Our research corroborates these findings, revealing a linear positive correlation between SIRI and survival outcome in ASCVD patients. This consistency suggests that SIRI holds significant potential as prognostic value for mortality from all causes in individuals with ASCVD.

Moreover, we discovered that serum uric acid partially mediated the link between SIRI and overall mortality among ASCVD individuals. This provides insight into the potential mechanisms that may underpin this connection. We detected positive correlation between SIRI and serum uric acid. Serum uric acid has been implicated in promoting atherosclerosis by activating inflammatory pathways, including AMP-activated protein kinase and phosphatidylinositol-3 kinase [40]. A mouse-based experimental study demonstrated that uric acid could exacerbate myocardial ischemia-reperfusion injury by increasing reactive oxygen species levels, which subsequently activate NOD-like receptor pyrin domain-containing protein 3 [41]. Oxidative stress contributes to the pathogenesis of atherosclerosis by inducing endothelial cell dysfunction and vasodilation, as well as promoting inflammation in macrophages and other immune cells, platelet aggregation, and LDL oxidation [40]. A comprehensive meta-analysis of 14 studies found that hyperuricemia significantly increases the risk of death from coronary heart disease, especially in women [42]. Additionally, research has demonstrated that uric acid levels were associated with ASCVD-related complications, including heart failure and acute coronary syndrome [43, 44]. These findings support the mediating role of serum uric acid in the connection between SIRI and mortality from all causes among ASCVD individuals, offering valuable insight into the underlying biological mechanisms.

This study features several notable strengths. It employs data from a nationally representative database, incorporating survey weighting methods to improve the generalizability of the findings. Importantly, this study is the first to use mediation analysis to detail how serum uric acid mediates the connection between SIRI and overall mortality in ASCVD individuals. This novel approach offers a foundational reference for further investigations into the potential mechanisms underlying this relationship. Moreover, to account for potential unmeasured confounders, we computed the *E*-value, which further affirms the robustness of our findings. Despite these strengths, the study also presents certain limitations that warrant attention. Primarily, it relies on baseline SIRI levels to assess their correlation with all-cause mortality, without considering the possible effects of dynamic changes in SIRI levels throughout the follow-up period. Furthermore, while the findings are based on an analysis of the UnitedStates population, additional research is required to ascertain the effectiveness of these findings to other countries, which may exhibit distinct epidemiological traits and health dynamics.

## 5. Conclusions

This study's results clearly indicate a connection between SIRI and all-cause mortality risk among ASCVD individuals. The findings are uniformly strong, supporting the reliability of SIRI in predicting mortality across different time points for ASCVD patients. Additionally, the study highlights the role of serum uric acid as mediator in the link between SIRI and all-cause mortality. Therefore, managing systemic inflammatory responses could be a crucial strategy for reducing mortality risk in the ASCVD patient population.

## Figures and Tables

**Table 1:** Baseline characteristic of study participants.

Characteristics	Survival participants	Dead participants	*p*-Value
Number of participants	2760	1933	—
Demographic variables	—	—	—
Age (years)	62.000 (52.000, 71.000)	75.000 (66.000, 80.000)	<0.001
Gender, *n* (%)	—	—	0.664
Male	1510 (54.745)	1178 (53.868)	—
Female	1250 (45.255)	755 (46.132)	—
Race/ethnicity, *n* (%)	—	—	<0.001
White	1338 (71.976)	1327 (82.406)	—
Black	611 (11.223)	313 (8.926)	—
Mexican	345 (5.126)	166 (2.940)	—
Other	466 (11.674)	127 (5.728)	—
Education, *n* (%)	—	—	<0.001
< High school	397 (7.738)	405 (15.249)	—
High school	1148 (40.649)	851 (46.037)	—
> High school	1215 (51.613)	677 (38.714)	—
PIR	2.470 (1.320, 4.310)	1.920 (1.230, 3.030)	<0.001
Healthy behavior factors	—	—	—
Smoking status, *n* (%)	—	—	<0.001
Nonsmokers	1114 (39.590)	699 (35.796)	—
Former smokers	1001 (36.115)	906 (45.812)	—
Current smokers	645 (24.295)	328 (18.392)	—
Alcohol consumption, *n* (%)	—	—	<0.001
Nondrinkers	353 (10.191)	333 (17.240)	—
Former drinkers	668 (21.874)	806 (39.283)	—
Current drinkers	1739 (67.935)	794 (43.476)	—
BMI (kg/m^2^)	29.680 (26.060, 34.000)	28.260 (25.070, 32.300)	<0.001
Laboratory parameter	—	—	—
Lymphocyte (10^9^/L)	2.000 (1.600, 2.500)	1.800 (1.300, 2.300)	<0.001
Monocyte (10^9^/L)	0.600 (0.500, 0.700)	0.600 (0.500, 0.700)	0.026
Neutrophil (10^9^/L)	4.200 (3.300, 5.200)	4.500 (3.500, 5.600)	<0.001
History of disease	—	—	—
T2D, *n* (%)	—	—	<0.001
No	1708 (66.857)	1107 (58.669)	—
Yes	1052 (33.143)	826 (41.331)	—
Hypertension, *n* (%)	—	—	<0.001
No	664 (28.109)	375 (19.542)	—
Yes	2096 (71.891)	1558 (80.458)	—
Hyperlipidemia, *n* (%)	—	—	0.17
No	347 (11.205)	275 (12.684)	—
Yes	2413 (88.795)	1658 (87.316)	—

Abbreviations: BMI, body mass index; PIR, poverty-income ratio; T2D, type 2 diabetes.

**Table 2:** Relationship between SIRI and all-cause mortality among atherosclerotic cardiovascular disease patients.

Characteristic	Model 1		Model 2		Model 3		Model 4	
	HR (95% CI)	*p* - Value	HR (95% CI)	*p* - Value	HR (95% CI)	*p* - Value	HR (95% CI)	*p* - Value
SIRI	—	—	—	—	—	—	—	—
Continuous	1.272 (1.192, 1.356)	< 0.001	1.199 (1.135, 1.267)	< 0.001	1.196 (1.137, 1.259)	< 0.001	1.192 (1.131, 1.256)	< 0.001
Quartiles	—	—	—	—	—	—	—	—
Q1	1	—	1	—	1	—	1	—
Q2	1.200 (0.975, 1.477)	0.086	1.114 (0.919, 1.351)	0.272	1.117 (0.926, 1.347)	0.247	1.090 (0.898, 1.323)	0.385
Q3	1.516 (1.273, 1.806)	< 0.001	1.264 (1.073, 1.489)	0.005	1.260 (1.068, 1.486)	0.006	1.227 (1.035, 1.454)	0.019
Q4	2.612 (2.205, 3.093)	< 0.001	1.903 (1.607, 2.253)	< 0.001	1.843 (1.562, 2.173)	< 0.001	1.810 (1.529, 2.143)	< 0.001
*p* for trend	—	< 0.001	—	< 0.001	—	< 0.001	—	< 0.001

*Note*: Model 1: Unadjusted covariates. Model 2: Adjusted for age, gender, race/ethnicity, education, and poverty-income ratio. Model 3: Adjusted for age, gender, race/ethnicity, education, and poverty-income ratio, smoking status, alcohol consumption, and body mass index. Model 4: Adjusted for age, gender, race/ethnicity, education, poverty-income ratio, smoking status, alcohol consumption, body mass index, hypertension, type 2 diabetes, and hyperlipidemia.

Abbreviations: CI, confidence interval; HR, hazard ratio; SIRI, systemic inflammatory response index.

## Data Availability

This study used data from a publicly available dataset. These data can be found at https://www.cdc.gov/nchs/nhanes.

## Supporting Information

Supporting Information Supporting Information. Figure S1: Features selection using the Boruta algorithm. Figure S2: Kaplan–Meier survival curves for all-cause mortality. Figure S3: Relationship between SIRI and all-cause mortality with RCS method. Figure S4: Time-dependent ROC curves and time-dependent AUC values of the SIRI for predicting all-cause mortality. Table S1: Variable's selection using univariate Cox models.
