# Supplementary material for: Dynamic and Static Effects of the Systemic Inflammatory Response Index on All-Cause Mortality in Individuals With Atherosclerotic Cardiovascular Disease: Evidence From National Health and Nutrition Examination Survey
Source: Mediators Inflamm. 2025 Apr 16;2025:5343213. doi: 10.1155/mi/5343213 (PMC12017944; doi:10.1155/mi/5343213)
Supplement: Supporting Information — Figure S1: Features selection using the Boruta algorithm. Figure S2: Kaplan–Meier survival curves for all-cause mortality. Figure S3: Relationship between SIRI and all-cause mortality with RCS method. Figure S4: Time-dependent ROC curves and time-dependent AUC values of the SIRI for predicting all-cause mortality. Table S1: Variable's selection using univariate Cox models. [file 5343213.f1.docx]

**Dynamic and static effects of the systemic inflammatory response index on all-cause mortality in individuals with atherosclerotic cardiovascular disease: Evidence from NHANES**

**Figures and Tables**

Figure S1 Features selection using the Boruta algorithm.

Figure S2 Kaplan-Meier survival curves for all-cause mortality.

Figure S3 Relationship between SIRI and all-cause mortality with RCS method.

Figure S4 Time-dependent ROC curves and time-dependent AUC values of the SIRI for predicting all-cause mortality.

Table S1. Variable’s selection using univariate Cox models.


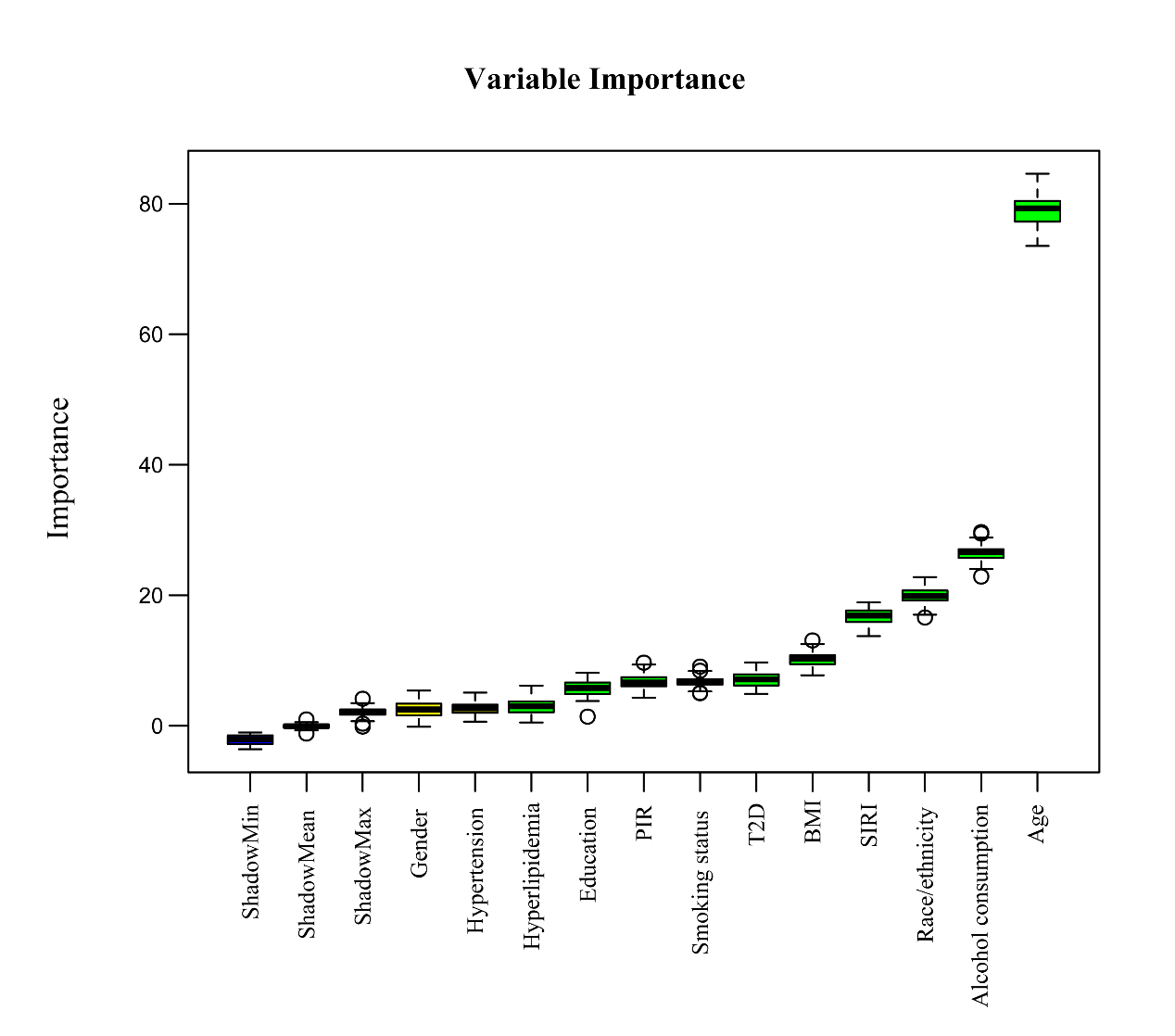


Figure S1. Features selection using the Boruta algorithm. PIR, Poverty-income ratio; T2D, Type 2 diabetes; BMI, Body mass index; SIRI, Systemic inflammatory response index.


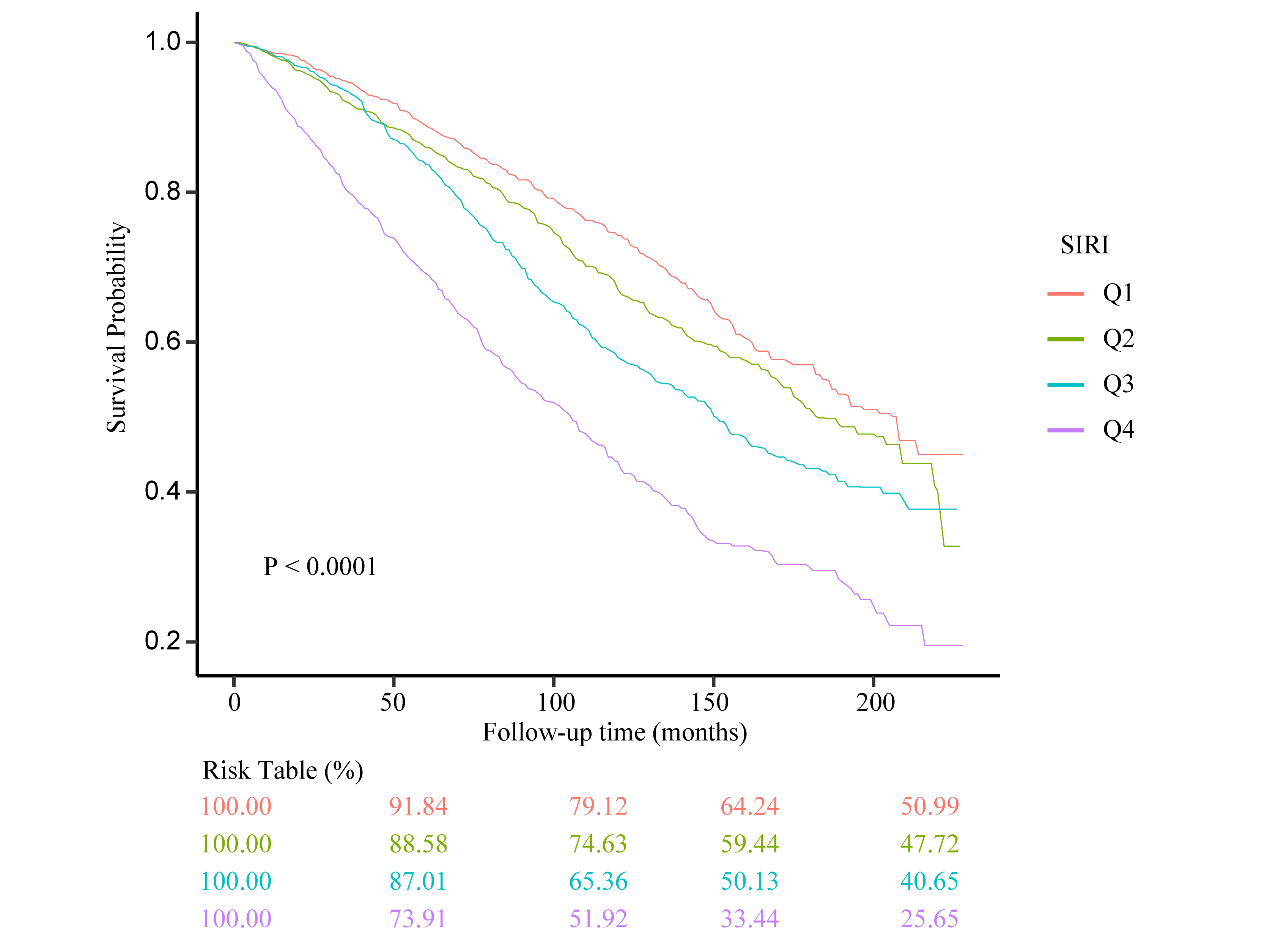


Figure S2. Kaplan-Meier survival curves for all-cause mortality. SIRI: Systemic inflammatory response index.


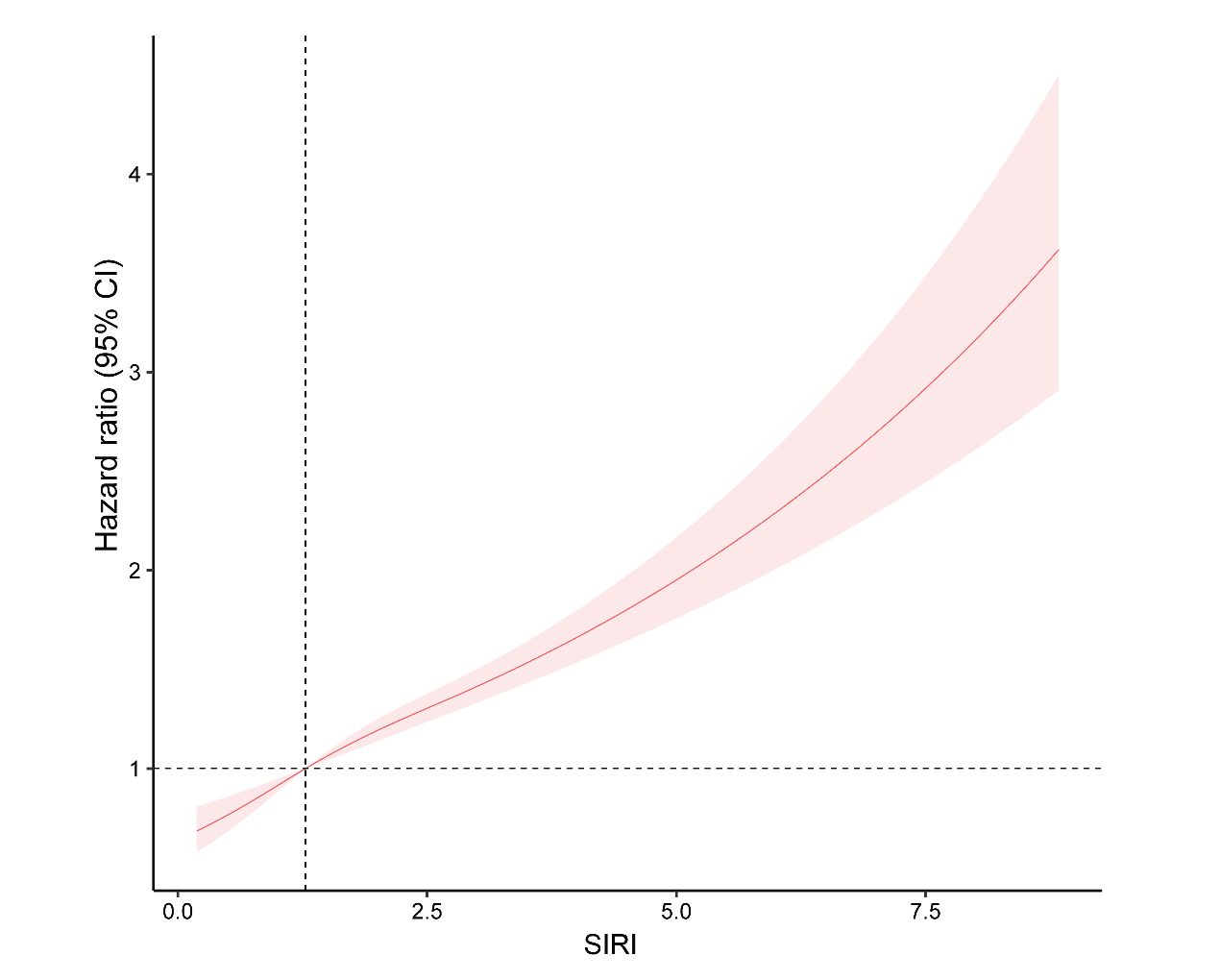


Figure S3. Relationship between SIRI and all-cause mortality with RCS method. SIRI: Systemic inflammatory response index. RCS: Restricted cubic spline.


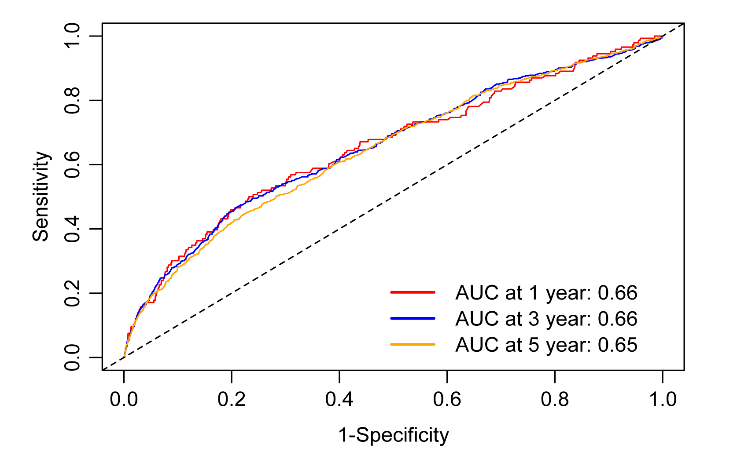


Figure S4. Time-dependent ROC curves and time-dependent AUC values of the SIRI for predicting all-cause mortality. ROC: Receiver operating characteristic; AUC: Area under curve; SIRI: Systemic inflammatory response index.

Table S1. Variable’s selection using univariate Cox models

| Characteristics | HR | 95%CI | *P-*value |
| --- | --- | --- | --- |
| Age | 1.080 | (1.072, 1.088) | < 0.0001 |
| Gender |  |  |  |
| Male | Reference |  |  |
| Female | 0.976 | (0.876, 1.088) | 0.665 |
| Race/ethnicity |  |  |  |
| White | Reference |  |  |
| Black | 0.749 | (0.647, 0.866) | < 0.0001 |
| Mexican | 0.586 | (0.457, 0.751) | < 0.0001 |
| Other | 0.601 | (0.446, 0.809) | < 0.001 |
| Education |  |  |  |
| < high school | Reference |  |  |
| High school | 0.748 | (0.628, 0.891) | 0.001 |
| > high school | 0.578 | (0.486, 0.689) | < 0.0001 |
| PIR | 0.853 | (0.821, 0.886) | < 0.0001 |
| Smoking status |  |  |  |
| Non-smokers | Reference |  |  |
| Former smokers | 1.288 | (1.124, 1.476) | < 0.001 |
| Current smokers | 0.821 | (0.692, 0.973) | 0.023 |
| Alcohol consumption |  |  |  |
| Non-drinkers | Reference |  |  |
| Former drinkers | 1.033 | (0.871, 1.225) | 0.707 |
| Current drinkers | 0.580 | (0.481, 0.701) | < 0.0001 |
| BMI | 0.975 | (0.965, 0.985) | < 0.0001 |
| T2D |  |  |  |
| No | Reference |  |  |
| Yes | 1.610 | (1.451, 1.787) | < 0.0001 |
| Hypertension |  |  |  |
| No | Reference |  |  |
| Yes | 1.574 | (1.359, 1.822) | < 0.0001 |
| Hyperlipidemia |  |  |  |
| No | Reference |  |  |
| Yes | 0.943 | (0.794, 1.119) | 0.500 |
